# The cognitive dysfunction of claustrum on Alzheimer’s disease: A mini-review

**DOI:** 10.3389/fnagi.2023.1109256

**Published:** 2023-04-14

**Authors:** Chun-Yan Chen, Guang-Yi Yang, Hai-Xia Tu, Xu-Chu Weng, Chun Hu, Hong-Yan Geng

**Affiliations:** ^1^Key Laboratory of Brain, Cognition and Education Science, Ministry of Education, South China Normal University, Guangzhou, China; ^2^Guangdong Key Laboratory of Mental Health and Cognitive Science, Institute for Brain Research and Rehabilitation, South China Normal University, Guangzhou, China

**Keywords:** claustrum, Alzheimer’s disease, cognitive dysfunction, neural circuits, pathology

## Abstract

Alzheimer’s disease (AD) is one of the most common neurodegenerative diseases characterized by cognitive deficits and dementia. AD entails predominant pathological characteristics including amyloid beta (Aβ) plaque formation, neurofibrillary entanglements, and brain atrophy, which gradually result in cognitive dysfunctions. Studies showed that these pathological changes are found in a myriad of brain structures, including the claustrum (CLA), a nucleus that penetrates deeply into the brain and is extensively interconnected to various brain structures. The CLA modulates many aspects of cognitive functions, with attention, executive function, visuospatial ability, language, and memory in particular. It is also implicated in multiple neuropsychiatric disorders, of which one worthy of particular attention is AD-related cognitive impairments. To inspire novel AD treatment strategies, this review has summarized the CLA functionality in discriminative cognitive dysfunctions in AD. And then propose an array of potential mechanisms that might contribute to the cognitive impairments caused by an abnormal CLA physiology. We advocate that the CLA might be a new promising therapeutic target in combination with existing anti-AD drugs and brain stimulation approaches for future AD treatment.

## Introduction

Alzheimer’s disease (AD) is a type of ubiquitous neurodegenerative disease characterized by cognitive dysfunction. This kind of neurodegenerative disease deprives individuals of the ability to concentrate and poses challenges to executive functions and can gradually progress to misplacing, narrative incompletion, and further result in delayed recollection as well as false memories in the later stage of AD (El Haj et al., 2020; Knopman et al., 2021). Previous studies identified a myriad of brain areas that are involved in AD development, such as the prefrontal cortex (PFC), entorhinal cortex (EC), and hippocampus. While recently, a nucleus located in the forebrain named claustrum (CLA), has gained increasing popularity due to its functions in attention, executive function and memory (Smith et al., 2020; Nikolenko et al., 2021; Whalley, 2022), which are perceived as an integral part of AD cognitive impairment.

**Figure 1:**
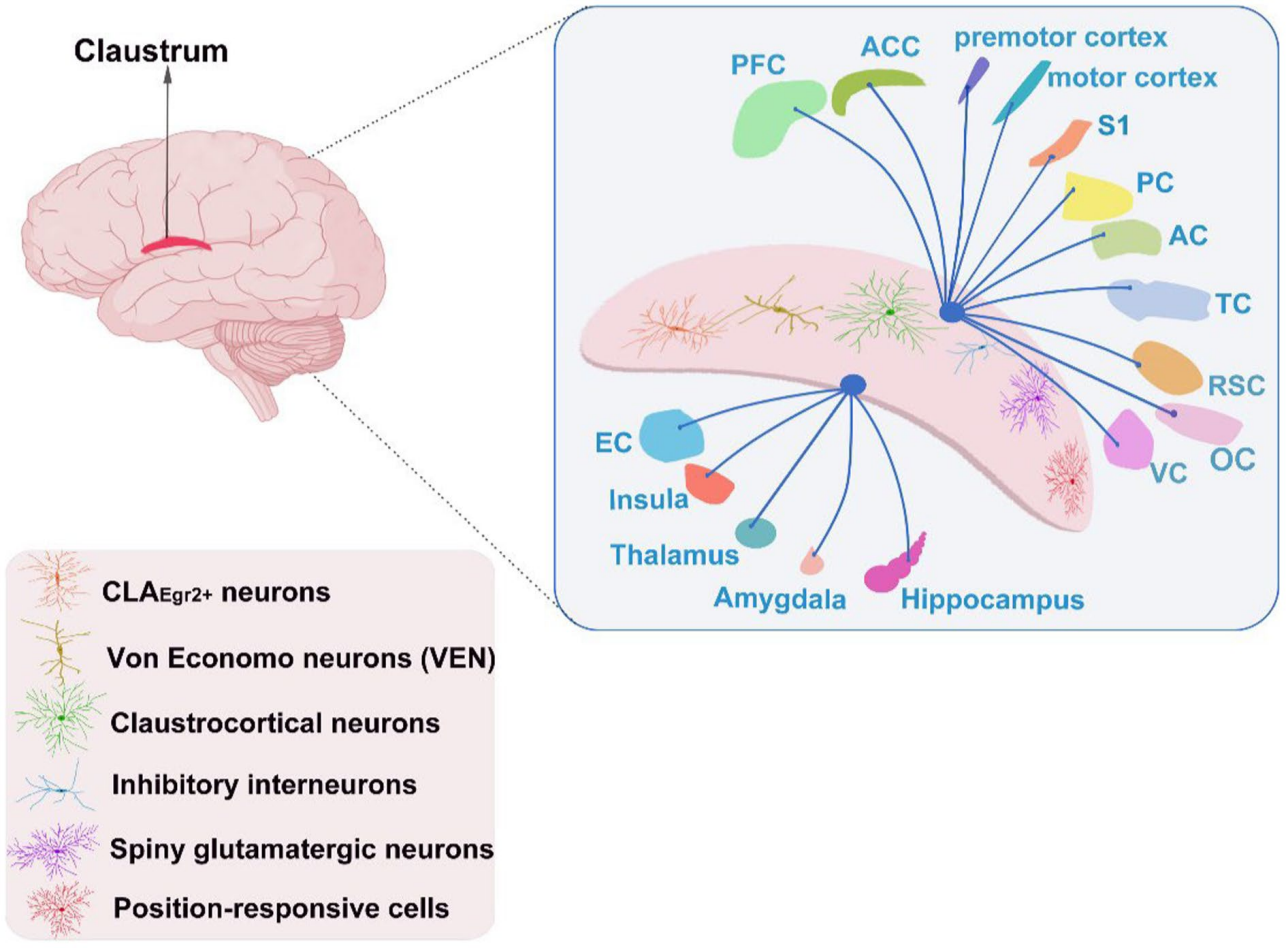
The cognitive neuronal types of the CLA and its interconnection with other brain areas. PFC, prefrontal cortex; ACC, anterior cingulate cortex; S1, primary somatosensory cortex; PC, parietal cortex; AC, auditory cortex; TC, temporal cortex; RSC, retrosplenial cortex; VC, visual cortex; EC, entorhinal cortex; OC, occipital cortex.

The CLA is a thin sheet of grey matter deeply penetrating into the forebrain and sandwiching between insula and putamen. It is widely interconnected to brain structures, e.g., PFC, anterior cingulate cortex (ACC), EC, hippocampus, amygdala, and insula. The CLA assembles substantial cognitive-relevant cells such as Von Economo neurons (VEN) and position-responsive cells apart from multitudinous claustral neurons (see Figure 1; Smythies et al., 2014; Jankowski and O'Mara, 2015; Smith et al., 2020). Consequently, the CLA and its related circuitries get involved in several cognitive functions, including attention, executive function (White et al., 2018), visuospatial ability (Gould et al., 2006), language (Van Rinsveld et al., 2017), and memory (Seo et al., 2016), and these cognitive functions achieved by varies brain networks in AD tend to denigrate over the course of the disease.

Prominent pathological alterations are observed in the CLA with its related circuits in AD patients. Both the plaques and the neurofibrillary tangles accumulate in the CLA (Braak and Braak, 1991; Thal et al., 2002). More clearly, the senile plaques exist in the third phase of Aβ deposition in the CLA (Thal et al., 2002), while the mild neurofibrillary tangles occur in the CLA at stage IV, with increasing severity in stage V and VI (Braak and Braak, 1991). AD patients accompanied with delusional symptoms possess a significant grey matter volume reduction in the left CLA (Bruen et al., 2008). Neuronal loss and synaptic pathology happening in the CLA are hallmarks of AD pathology, particularly in the anterior portion (Baloyannis et al., 2013). At the circuit level, the paramygdala part of the CLA connected to the entorhinal cortex suffers the primary deterioration in AD brains (Morys et al., 1996).

This review illustrates the functions of CLA in various cognitive impairments of AD, respectively. And it further elucidates the underlying mechanisms by combining CLA itself with its relevant circuitries in modulating pathological changes of AD in five cognitive perspectives: attention, executive function, visuospatial ability, language, and memory, to demonstrate the feasibility of targeting CLA for future treatment of AD cognitive dysfunction.

## Attention

Attention is the first affected non-memory domain in AD. Clinically, at the early stage where there is no or little memory deficit, AD patients are frequently muddled and unable to concentrate on tasks that are effortless to accomplish previously (Schumacher et al., 2019). And they had difficulty in processing attentional information with higher reaction speed and error rate when switching tasks (Hennawy et al., 2019). In mild cognitive impairment (MCI), a neurodegenerative disease indicated an incremental risk for evolving to AD, the functional connectivity of the CLA has increased within the salience network (SN), a brain network highly involved in mediating the attention function of AD (Schultz et al., 2017).

The CLA enables to mediate attention at both cellular and circuit levels. The activities of abundant VEN in CLA promote the interaction between the default mode network (DMN) and the task-related network in attention (Smythies et al., 2014). The CLA has the resilience to distraction when chronic and acute inactivation on claustral Egr2-expressing neurons (CLA_Egr2+_) in two-alternative forced-choice behavioral tasks by presenting irrelevant auditory distractor simultaneously to mice, which is attributed to the activation of CLA_Egr2+_ neurons modulated cortical sensory processing and suppression on tone representation of the auditory cortex (Atlan et al., 2018). Genetically-assisted silencing of CLA neurons delayed the acquisition of conditioned responses, suggesting that the CLA is essential in acquiring classical conditioning tasks, mainly in attentional processes concerning conditioned/unconditioned stimulus association (Reus-García et al., 2021). Across the claustral pathways, neurons projecting to ACC are more densely and evenly distributed than those to the primary somatosensory cortex (S1), implicating that the CLA may preferentially coordinate attention-relevant functions regulated by ACC (White et al., 2017). An attentional strategy demonstrates that PFC-CLA is mainly achieved through feedforward inhibition imposed by CLA on cortical patterns. These evidences support this hypothesis that the CLA integrates limbic information from the medial PFC, thalamus and amygdala to direct attention relevant sensory events in modality-related areas of motor and sensory cortex (Smith et al., 2019). Although there is a range of evidence supporting the role of CLA in mediating attention at cellular and circuit levels, the neurobiological basis of the attentional deficits in AD remains unclear, yet it is reasonable to assume that the lesions of the CLA occurring at the early stages of AD possibly affect attentional regulation.

## Executive function

Executive function is the ability to control or direct behavior from the top to down, like decisions making and motive initiation (Schumacher et al., 2019; Gaubert et al., 2022). Executive dysfunction occurs in early stages of AD (Tort-Merino et al., 2022), and it highly corresponds to the decreasing volumes of central executive network including lateral parietal cortex, dorsolateral frontal cortex and partial premotor area which have dense connections with CLA (Smythies et al., 2014; Miro-Padilla et al., 2020; Fang et al., 2021). It is demonstrated that a negative correlation between the Reading the Mind in the Eyes Test concerning executive function and the functional connectivity of the SN in the CLA in the early stage of AD (Valera-Bermejo et al., 2021). In human imaging, the left CLA was activated in the executive tests covering Stroop, N-back, and Go/No Go (Minzenberg et al., 2009). Likewise, the CLA is engaged in the underlying processes of executive function, i.e., the activation occurs during both the switching and updating tasks (McKenna et al., 2017).

According to a series of studies, together CLA with its pathways is substantiated to affect executive function. A higher error rate of behavioral flexibility shows in rats during the reversal of the excitotoxic anterior CLA group in a water-maze experiment compared to the control (Grasby and Talk, 2013). The cognitive control of action is further uncovered from CLA by manipulating CLA projection neurons during 5-choice serial reaction time task employing optogenetic modulation on claustral Gnb4-cre mice (White et al., 2020). Besides, the claustral circuits mediate executive performance. The claustral spiny glutamatergic neurons and inhibitory interneurons are monosynaptically innervated by the ACC, the former of which has magnified ACC inputs in a way that is suppressed by claustral inhibitory microcircuits, which demonstrates ACC-CLA as a modulator in top-down action control (White et al., 2018). Chemogenetically activating or inhibiting the CLA-PFC, respectively, would intensify or attenuate the impulsive-like behaviors in 5-choice serial reaction time task (Liu et al., 2019), whereas chemogenetic inhibition on the bilateral claustrocortical neurons projecting to S1 decreases the inappropriate lick response (Chevée et al., 2022). Given that CLA suffers a certain executive dysfunction with neuroimaging analysis in the early stage of AD, future attempts might be made to alleviate executive function symptoms in AD by activating CLA and its related cortical circuits.

## Visuospatial ability

Patients with AD initially have clinical challenges with visuospatial difficulties, such as spatial disorientation, being trapped in familiar surroundings (Quental et al., 2009). Additionally, AD patients get stuck in face discrimination and struggling to process complicated visual scenes (Quental et al., 2009; Knopman et al., 2021). In an imaging study, the grey matter density fluctuation in the left CLA of AD patients corresponds to scores changes in the visuo-constructional apraxia test (Venneri et al., 2008). The AD patients further suffered claustral inactivity during the visuospatial paired information encoding and retrieval (Gould et al., 2006).

Evidence has identified several possible mechanisms of the CLA in mediating visuospatial disorders in AD. Initially, abundant place-responsive cells with hippocampal and EC characteristics have been observed in the anterior CLA in mice. They display rapid spatial activity when exposed to the environment (Jankowski and O'Mara, 2015). Nevertheless, the lewy body pathology in CLA leads to decreasing neuronal activity and even atrophy in this area, thereby disrupting its spatial response function which has been detected in AD patients with alpha-synuclein immunohistochemistry (Hashimoto and Masliah, 1999). In addition, the impaired integration function of CLA circuits induces visuospatial dysfunction in global aspects. Compared with auditory, somatosensory, or motor areas, the lewy body pathology in the CLA is more closely associated with visual areas, and the damage of the visuo-claustral pathway that connects with insula and EC would result in visual misidentification (Yamamoto et al., 2007). Meanwhile, the CLA has extensive connections with both the hippocampus and EC (Smythies et al., 2014; Smith et al., 2020), and the claustral glutamatergic neurons projecting to limbic cortex were activated during sleep, which powerfully reinforces the function of the CLA in space and navigation (Luppi et al., 2017). Furthermore, it interconnects with the occipital, temporo-parietal, and frontal cortices, a brain network involved in spatial and visual constructive abilities, which can be associated with early mechanisms of cognitive deterioration in the progression to AD (Smythies et al., 2014; Plaza-Rosales et al., 2023). The anatomical changes in the CLA give rise to compromised visuospatial functionality of AD. All this points to CLA as a crucial spot in coordination concerning visuospatial dysfunction owing to its place-responsive cells and interconnections with numerous brain structures in AD patients.

## Language

Language dysfunction appears in early AD, showing preliminary difficulty in semantic abilities (Venneri et al., 2008). Mild AD patients are diagnosed to be subject to language obstacles in verbal fluency, auditory perception, reading comprehension, and narrative performance (Tsantali et al., 2013). In a study of nicotinic acetylcholine receptor binding in the preclinical patient group, verbal memory learning in MCI patients was found to be associated with discrete uptake reduction in CLA, which provides evidence that the left CLA might modulate cognitive performance in diagnosed or prodromal AD (Terrière et al., 2010). The semantic abilities deteriorate in the early stage of AD, whereas the volumetric changes in bilateral CLA of AD patients were found prominently associated with confrontational naming tasks and categorization fluency (Venneri et al., 2008).

A series of neuroimaging investigations prove that CLA occupies an essential linguistic role. Combined with fMRI on the brains of proficient bilingual subjects doing simple and complex addition mental arithmetic tasks, the CLA has different levels of evocation in people with different language dominance (Van Rinsveld et al., 2017). The MRI scans of all aphasia patients emerge ischemic lesions in the left hemisphere, and the largest areas of overlapping foci are localized in the CLA and other brain structures (Marangolo et al., 2014). By conducting meta-analysis, the right CLA, bilateral inferior frontal cortex and superior temporal gyrus performed a clear consistent neural motivation pattern in written language and speech processing in the child group (Zhu et al., 2014), while it also engaged in two of thirteen major clusters of insula connections of language function (Ardila et al., 2014). The medial CLA provides a robust contralateral connection between the right subcortex and left PFC, resulting in patients with right subcortical lesions performing worse than the left in cognitive linguistic functions (Girija et al., 2018). Altogether, the language function is obviously modulated by CLA itself and its circuit connections. Thus, an intervention targeting CLA could potentially salvage linguistic dysfunction in AD patients.

## Memory

The most severely damaged cognitive deficit in AD patients is mnemonic dysfunction, where they have difficulty not only in the encoding and storage stages but also in the retrieval stage (El Haj et al., 2020). They easily forget familiar faces or things since their brains fail to integrate these memories (Roy et al., 2016). Notable evidence manifests amnesia AD patients have defective functions in episodic memory (Schwindt and Black, 2009), working memory (Stopford et al., 2012), and contextual memory (El Haj et al., 2020). During memory encoding and retrieval paradigms, the CLA exhibits higher activity in healthy controls than in AD patients (Schwindt and Black, 2009). Resting-state fMRI notes that AD patients have weakened functional connectivity between the right CLA and the amygdala, which elucidates the relevancy to memory deficits (Wang et al., 2016).

The CLA has a substantial effect on memory. The neurons of anterior CLA modulate theta rhythm critical to episodic memory impairment in early AD, which requires the synchronized activity of CLA and relevant cortical regions (Jankowski and O'Mara, 2015). The CLA participated in acquiring stable long-term memory for the value of objects in a high-capacity fMRI study (Ghazizadeh et al., 2018). A hypothesis proposes that pathological loss of the VEN in the CLA attenuates task-related brain network functions in the CLA, especially memory functions in AD (Smythies et al., 2014). The posterior CLA projects onto the retrosplenial cortex (RSC), a well-established cortex in mnemonic processing regarding auditory cues, illuminating that the CLA-RSC has a significant influence on the function of remote memory retrieval in rodents (Todd et al., 2016). The CLA-medial EC is activated by new contexts and enables to modulate the function of the medial EC (Kitanishi and Matsuo, 2017), which may in turn influence contextual memory in AD patients. The CLA is further capable of processing working memory by means of its ipsilateral and contralateral connections with PFC, premotor, and motor areas (Smythies et al., 2014; Smith et al., 2020). And this is consistent with the postulation of Gattass et al., that the CLA is the gateway for perceptual information into the memory system, due to its extensive interconnectivity with almost the entire neocortex and its projections to the hippocampus, amygdala and basal ganglia (Gattass et al., 2014). The CLA deficits in AD patients are likely to be interposed in the development of several forms of memory impairments, supposing that intervention in the claustral neurons and CLA memory-related circuits probably adjust memory dysfunction in AD patients.

## Future direction

**Figure 2:**
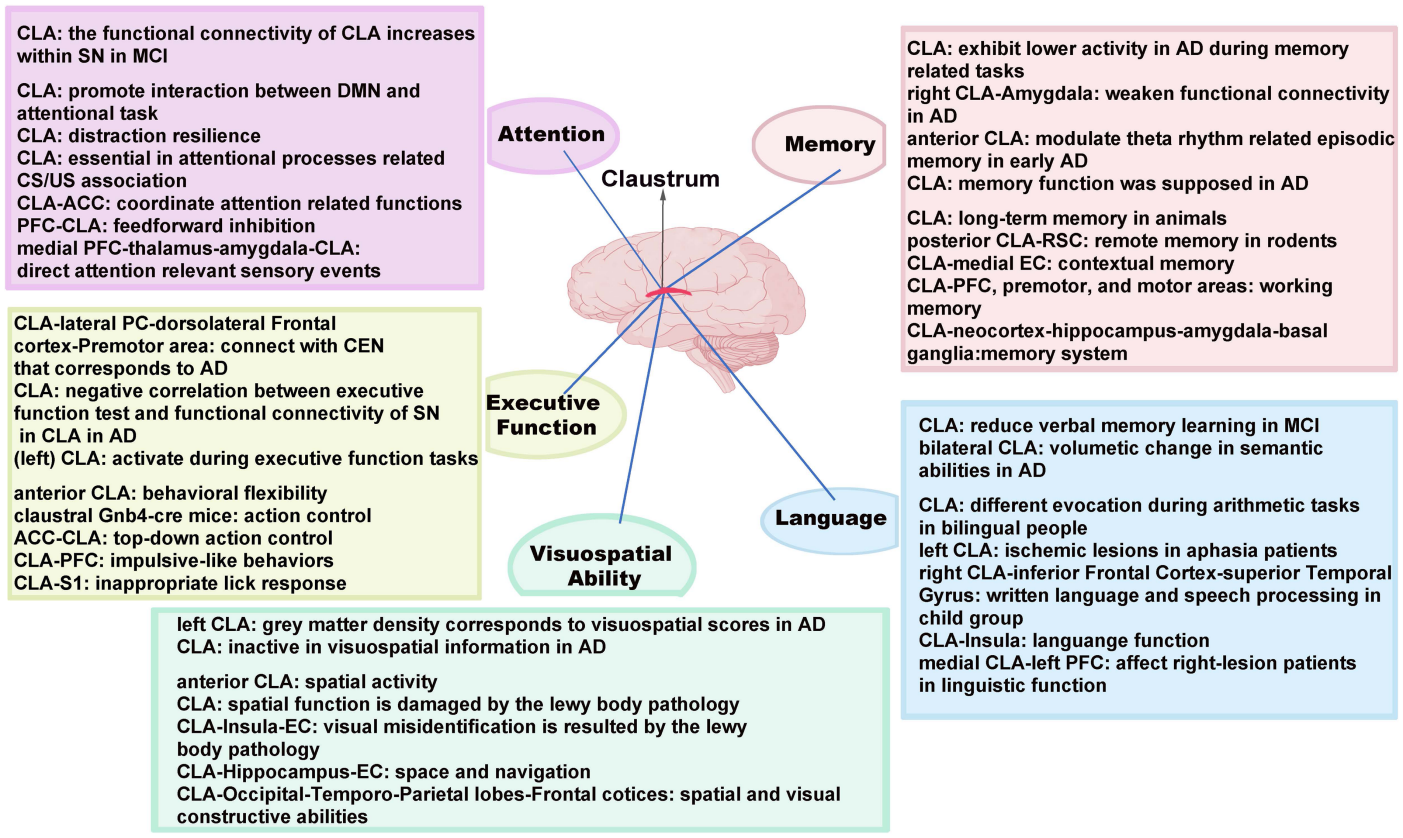
Major cognitive functions and the underlying mechanisms mediated by the CLA with its related circuits in AD. SN, salience network; MCI, mild cognitive impairment; DMN, default mode network; CS, conditioned stimulus; US, unconditioned stimulus; CEN, central executive network; ACC, anterior cingulate cortex; PFC, prefrontal cortex; S1, primary somatosensory cortex; PC, parietal cortex; EC, entorhinal cortex; RSC, retrosplenial cortex.

The evidence mentioned above provides an exhaustive account of the relationship between CLA and AD pathology in terms of cognitive functions, including attention, executive function, visuospatial skills, language, and memory (see Figure 2). It has analyzed and summarized the underlying mechanisms by which the CLA and claustral circuits might mediate AD pathological changes in terms of these cognitive functions. Although the CLA has the capability of multimodal information integration and is involved in regulating high-order cognition, more basic researches are required to clarify the relationship between the CLA and AD *via* advanced structural and functional research techniques.

The CLA has widespread interconnections to various brain structures, with a structural basis for the integration of various cognitive functions. In detail, the respective interconnections between CLA and ACC, PFC are engaged in attention and executive function, while the CLA-PFC further regulates language and working memory and the CLA-S1 takes part in executive functions. For visuospatial ability, there are significant connections between the CLA and the visual cortex, insula, hippocampus, EC as well as temporal–parietal lobes. Similarly, the language can be modulated by the pathways of CLA and the inferior frontal gyrus, insula. It is also noted that multiple memories can be mediated by claustral circuits, in which CLA interconnects with a myriad of areas, including RSC, EC, premotor, and motor cortex. Although the CLA has the capability of multimodal information integration and is involved in regulating high-order cognition, the functions of these circuits in mediating distinct cognitive aspects in AD remains to be explored.

Some symptoms of the early stage of AD, such as attention deficit, executive dysfunction, language misinterpretation, and memory impairment, have been found to be associated with early pathological changes in CLA (Venneri et al., 2008; Schwindt and Black, 2009; Valera-Bermejo et al., 2021). Therefore, the therapeutic interventions on the CLA and claustral circuits may alleviate the progression of AD at early stage. For example, the chronic intracerebroventricular administration of AT IV receptors agonists, like norleucine1-Ang IV, remarkably improve the acquisition of spatial memory in AD mice (Prakash et al., 2015), while the high density of AT IV receptors was found in CLA (Chai et al., 2000). Thus, the AT IV receptor agonists in the CLA could serve as a promising target for drug intervention to alleviate spatial memory impairment of AD. It is also found that the left CLA increases glucose metabolism in AD in both one-year and one-month deep brain stimulation (DBS; Laxton et al., 2010), which illustrates the significance of CLA for the improvement of cognitive symptoms in AD. The CLA, meanwhile, is involved in several brain networks that regulate various cognitive functions in AD patients. Considering the potential claustral mechanism in AD, it should be a promising approach to administer drugs or DBS activation to the CLA with its related circuits.

Furthermore, it certainly requires more basic pathological and physiological studies to elucidate the function of CLA in mediating AD. Although many types of cognitive cells that have been identified so far, there might be yet other unknown neurons carrying utterly different cognitive functions. Additionally, despite the great number of circuitries being discerned, the function of specific circuits remains to be resolved, especially that are profoundly implicated in AD etiology. Besides, animal models that could be implemented in AD-CLA studies are still lacking. Given that the CLA and the claustral pathways robustly intermediate multiple cognitive functions, it would be a promising direction for future research to simultaneously monitor the activity of the CLA with its brain networks in AD.

## Conclusion

This review highlights a fundamental but previously overlooked brain region, the CLA, and elaborately demonstrates its cognitive function on attention, executive function, visuospatial ability, language, and memory in AD. The claustral pathological changes are often found in structural and functional neuroimaging studies in AD, while the underlying mechanisms behind it are rarely analyzed, or even interpreted in terms of higher-order cognitive functions. We have combined normal physiological functions of the CLA and its pathological changes in AD to provide preliminary insights on the inferential framework of pathogenic mechanisms and attempted to propose certain therapeutic strategies for early-stage AD treatment by targeting CLA with its related circuits.

## Author contributions

C-YC and H-YG designed this review framework. C-YC, G-YY, H-XT, and H-YG searched the relevant literature. C-YC, G-YY, H-XT, X-CW, CH, and H-YG wrote the manuscript. All authors contributed to the article and approved the submitted version.

## Funding

This work was supported by grants from the Key-Area Research and Development Program of Guangdong province (2019B030335001), the National Natural Science Foundation of China (32200815), the National Social Science Foundation of China (20&ZD296), and the China Postdoctoral Science Foundation (2022M721218).

## Conflict of interest

The authors declare that the research was conducted in the absence of any commercial or financial relationships that could be construed as a potential conflict of interest.

## Publisher’s note

All claims expressed in this article are solely those of the authors and do not necessarily represent those of their affiliated organizations, or those of the publisher, the editors and the reviewers. Any product that may be evaluated in this article, or claim that may be made by its manufacturer, is not guaranteed or endorsed by the publisher.
